# Coding Schemes in the Archerfish Optic Tectum

**DOI:** 10.3389/fncir.2018.00018

**Published:** 2018-03-06

**Authors:** Adam Reichenthal, Mor Ben-Tov, Ronen Segev

**Affiliations:** ^1^Life Sciences Department and Zlotowski Center for Neuroscience, Ben Gurion University of the Negev, Beersheba, Israel; ^2^Department of Neurobiology, Duke University, Durham, NC, United States

**Keywords:** spike-triggered covariance, white noise analysis, linear-nonlinear models, receptive field, optic tectum

## Abstract

Many studies have yielded valuable knowledge on the early visual system but it is biased since the studies have focused on terrestrial mammals alone. Here, to better account for visual systems in different environments and animal classes, we studied the structure of early visual processing in the archerfish which harnesses its extreme visual ability to hunt by shooting water jets at prey hanging on vegetation above the water. Thus, the archerfish provides a unique opportunity to study visual processing in a vertebrate which is an expert vision-guided predator with a very different brain structure than mammals. The receptive field structures in the archerfish (both sexes) optic tectum, the main visual processing region in the fish brain, were measured and linear non-linear cascades were used to analyze their properties. The findings indicate that the spatial receptive field structures lie on a continuum between circular and elliptical shapes. In addition, the cells' functional properties display a richness of response characteristics, since many cells could be captured by more than a single linear filter. Finally, the non-linear response functions that link linear filters and neuronal responses were found to be similar to the non-linear functions of models that describe terrestrial mammalian single cell activity. Overall our results help to better understand the early visual processing system across vertebrates.

## Introduction

One of the key unresolved questions in neuroscience is how natural scene statistics influence the information flow in the early visual system. In mammals, influential theoretical frameworks have demonstrated that under simple assumptions that require the representation of terrestrial natural scene statistics to be efficient (Barlow, 1961), a set of filters that resemble receptive fields of simple cells in the visual cortex can be derived (Field, 1987; Olshausen and Field, 1996; van Hateren and van der Schaaf, 1998). However, detailed comparisons of the predictions of these theories to experimental data have shown that these theories fail to accurately replicate the internal structure of the receptive fields (Ringach, 2002). A possible approach to better understand the interplay between natural scene statistics and information processing in the visual system, is to investigate vertebrates that have evolved in an environment with a different statistical structure, namely an aquatic environment (Balboa and Grzywacz, 2003).

To address these issues, we used the archerfish as an animal model since it is an expert visual predator with a visual system that can process both underwater and land habitats. The archerfish hunts terrestrial insects above the water's surface by shooting powerful, accurate water jets in their direction that cause them to fall into the water (Lüling, 1958, 1963; Timmermans, 2000; Segev et al., 2007; Mokeichev et al., 2010; Ben-Simon et al., 2012; Tsvilling et al., 2012; Gabay et al., 2013; Pinsky et al., 2015). Thus, the archerfish's visual system also needs to process visual land environments, but its brain structure (Karoubi et al., 2016) is similar to the brain structure of many other teleosts which have adapted exclusively to the processing of underwater environments. Therefore, the archerfish's visual system provides a unique opportunity to understand the principles that shape the visual system information flow.

The central visual processing unit in the fish brain is the optic tectum (Northmore, 2011) which receives information directly from the retina on the opposite side. In addition, the visual layers of the optic tectum receive information from several pretectal areas which receive information from the retina and then relay it to the optic tectum. The cellular organization of the optic tectum appears to be uniform since cells are not organized into columnar structures. In the archerfish (Ben-Tov et al., 2013), goldfish (Maximov et al., 2005), and zebrafish (Niell and Smith, 2005; Johnston and Lagnado, 2012) the visual receptive fields of cells in the optic tectum have been classified into three categories: orientation-tuned cells, direction-tuned cells, and direction-agnostic cells. In addition, deeper, non-visual layers of the optic tectum receive information from the somatosensory, auditory, and lateral-line sensory systems which are mapped over the tectum cortex in a topographical manner.

There is a general consensus that in all classes of vertebrates the optic tectum is homologous to the mammalian superior colliculus. They are critical in determining the selection of gaze direction, as was shown in the monkey (Cowie and Robinson, 1994; Freedman et al., 1996), barn owl (du Lac and Knudsen, 1990), and goldfish (Herrero et al., 1998). However, similar to the saliency map found in the primary visual cortex of mammals (Zhaoping, 2016), the neural correlates of the saliency map exist in the archerfish optic tectum, which might help account for the existence of pop-out visual search in this animal (Ben-Tov et al., 2015). Therefore, when considering the functionality of different brain regions, the mammalian brain structure analogous to the fish optic tectum might actually be the primary visual cortex since these two brain regions are the primary visual areas of their respective lineages.

As described below, white noise analysis was used to study the spatiotemporal structure of the receptive field of the archerfish optic tectum. The discussion centers on the ways in which these observations can lead to a better understanding of information processing in the early visual system.

## Materials and methods

### Ethics statement

All experiments were approved by the Ben-Gurion University of the Negev Institutional Animal Care and Use Committee and were in accordance with government regulations of the State of Israel.

### Animals

**Figure 1:**
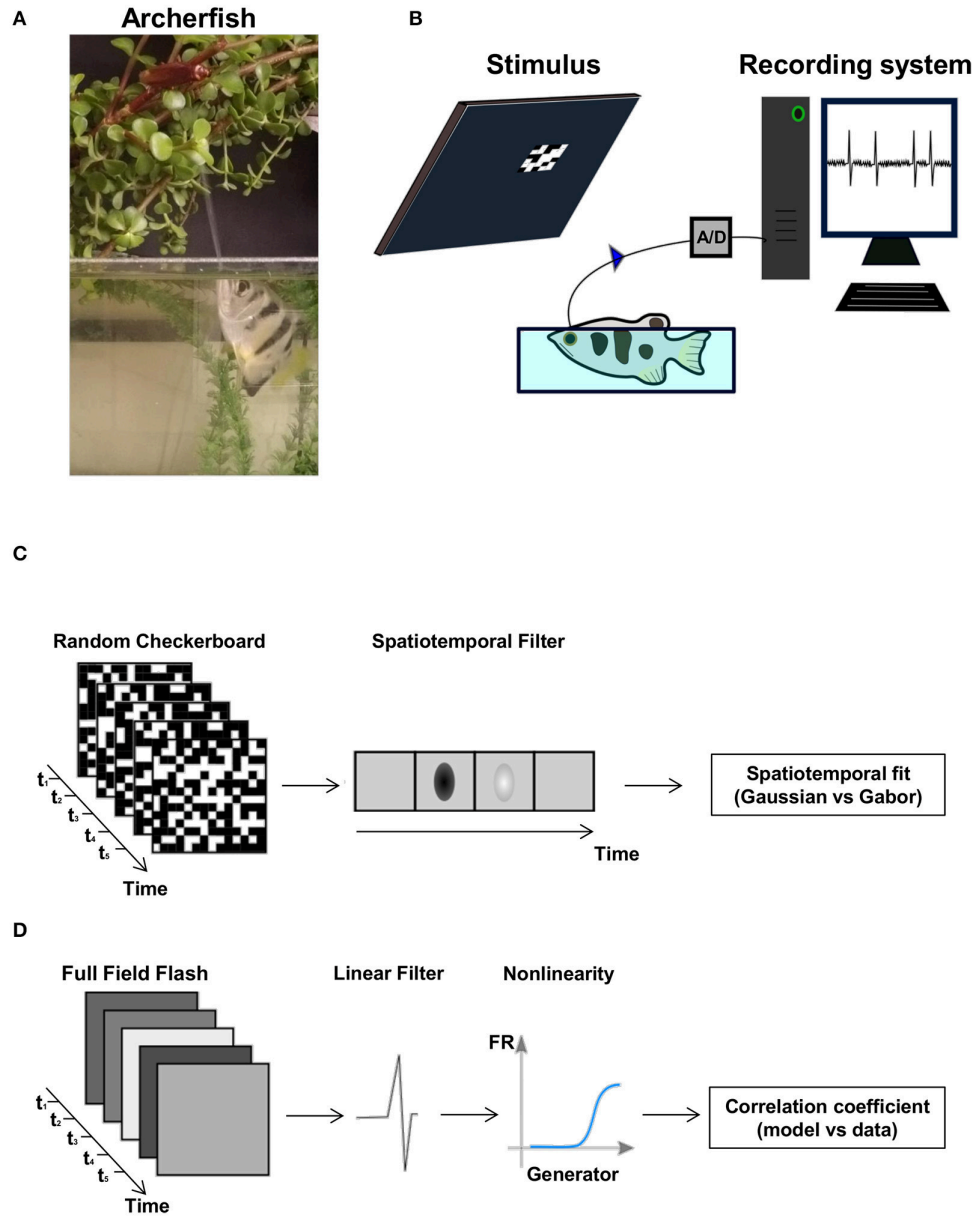
The archerfish and the experimental setup. **(A)** An archerfish hunting an insect above water level by shooting a powerful, accurate jet of water at it. **(B)** Overview of the recording setup. The fish is placed in a small water tank and a stimulus is presented to it on a computer screen. The spikes were recorded with an extracellular electrode. **(C,D)** The two types of visual stimuli used in the experiment: a random checkerboard stimulus where the value of each checker was drawn from a binary distribution and a full field diffuse flash where each light level was drawn from a Gaussian distribution and presented on the screen. Both stimuli were presented at a frame rate of 60 frames per second.

Acute experiments were performed on 15 archerfish (Toxotes Chatareus; Figure 1A), 6–14 cm in length, from both sexes. The fish were caught in the wild and purchased from a local animal distributor. The fish were housed in a water tank measuring 50 × 60 × 35 cm (~100 l) containing 3–12 fish, filled with brackish water (2–2.5 g of red sea salt mix per 1 l of water) at 26–28°C. The room was illuminated with artificial light on a 12:12-h day-night cycle.

### Surgery

Prior to surgery, the fish were anesthetized in a bath that contained MS-222 (A-5040, Sigma-Aldrich) at a concentration of 200 mg per liter of tank water. To control the pH level due to MS-222 acidity, NaHCO_3_ (S-5761, Sigma-Aldrich) at a concentration of 400 mg per liter of tank water was added to the bath. After the fish lost its buoyancy balance and flipped onto its back, it was placed in a restraining fish holder device and connected to a respiratory system containing a lower concentration of MS-222 (100 mg per liter of tank water and 200 mg of NaHCO_3_ per liter of tank water). During surgery the gills were continuously watered by a tube inserted into the fish's mouth to avoid possible respiratory failure from exposure to MS-222. Lidocaine (L-7757, Sigma) was applied to the scales above the brain region. Using a scalpel, the fish scales, skin and fatty tissue were removed, and Lidocaine was applied again on the wound as local anesthesia. At this stage 10–15 μl of the non-depolarizing muscle relaxant gallamine triethiodide (17g/l; G 8134, Sigma) was injected into the spine toward the tail. Then an opening in the skull above the optic tectum was made using a dental drill and the optic tectum was exposed. The optic tectum was covered with a thin layer of agarose to prevent dehydration and contamination. To reduce motion during experiment due to respiration, the skull was stabilized by attaching its frontal side with dental cement (GC Fuji PLUS, 001409, GC CORPORATION, Japan) to the water tank. During recording, anesthesia was removed by replacing the water of the respiratory system with fresh brackish water. We let the water tank to fill until the water level was up to 0.5 cm above eye level (Figure 1B). We kept the fish's gills watered through the tube during recording.

### *In vivo* electrophysiological recording

Extracellular action potentials were recorded with a sampling rate of 20 kHz by a single electrode (2–4 *MΩ*) mounted on a calibrated manipulator (Narishige, Tokyo, Japan). The electrode was inserted vertically to the optic tectum. The recordings were performed from all horizontal parts of the SWGZ layer (Karoubi et al., 2016), the superficial layer of the optic tectum, in depth of 300–500 μm. The signal was magnified (*x*10^4^) and filtered (band-pass box filter, 300 Hz−10 kHz range) by an amplifier (DAM 50, WPI) and then recorded using an in-house LabView program. The average duration of a typical recording session was 10 h, and we were able to hold single units up to 90 min.

### Electrode positioning and receptive field boundary outlining

While illuminating the fish's eye with an On-Off stimulus (1 s of white screen followed by 1 s of black screen in a repeating loop), we lowered the electrode until action potentials were detected. Then we gently tuned the electrode position to receive the strongest signal. To detect the limits of the cell's receptive field on the monitor, we manually moved a white bar across the screen in different orientations and directions until a strong reaction occurred; this line was marked as one of the edges of the receptive field. In a similar way, the bar was moved in different directions to determine the other edges of the receptive field. This method served to mark the receptive field boundaries to position the stimuli.

### Visual stimuli

Two interleaved types of stimuli, Random Checkerboard and Gaussian Full Field Flash, were presented on a monitor (Benq, model VW2245-T) at a refresh rate of 60 Hz (Figures 1C,D). Each minute of both types of stimuli was composed of two parts: 40 (or 50) s of non-repeated stimulus and 20 (or 10) s of repeated stimulus.

#### Random checkerboard stimulus

Checkerboard checkers randomly flickered white or black (Figure 1C). The range of checker size as projected onto the retina was 33–105 micron (mean 50 micron, STD = 17 micron) depending on the distance of the eye from the screen. This stimulus detected the spatiotemporal features of the cell. The reason for using a binary white noise of black and white checkers, rather than using a random checkerboard where each checker is flickering with Gaussian white noise is to reduce stimulus dimensionality. We found that otherwise the analysis become infeasible.

#### Gaussian full field flash stimulus

This stimulus was composed of full field flashes taken from a gray scale Gaussian distribution (Figure 1D). Since this stimulus had a one spatial dimension, its purpose was to detect the temporal features of the cell.

### Spike sorting

Spike sorting of single units was conducted offline using an in-house Matlab program. First, the signal was filtered with a band-pass filter (300–5000 Hz). Putative spikes were defined as events where the filtered signal crossed a threshold of 3.5 times the standard deviation of the signal. For each peak, 1.5 ms of signal before and after were preserved (for a total interval of 3 ms). Second, using a graphical user interface, events that did not have the shape of a spike or had impulse intervals shorter than an absolute refractory period were removed. The spikes were then clustered into one or more groups based on the spike's amplitude and width. Typically we identified one neuron per recording.

### Spatiotemporal analysis

The spatiotemporal receptive fields *RF*(*x, y*, τ) of the cells were obtained by calculating the spatial spike-triggered average (STA) at seven time steps (16.7 ms latency each) before the spike. To analyze the spatiotemporal profile of the receptive fields, two spatial dimensional Gaussian and Gabor functions were fitted to the data under the assumption that it was a separable receptive field:

(1) $$RF\left(x,y,\tau \right)=RF_{s}\left(x,y\right)RF_{t}\left(\tau \right)$$

Where *RF*_*s*_(*x, y*) and *RF*_*t*_(τ) capture the spatial and temporal components of the receptive field respectively. Prior to the fit, the original coordinate system (*x*′, *y*′) was translated by (*x*_0_, *y*_0_) and rotated by θ′ to align the receptive field's major axis with the vertical axis:

(2) $$x=(x^{\prime }-x_{0})\;\cos\;\theta^{\prime }+\left(y^{\prime }-y_{0}\right)\text{ sin }\theta^{\prime }$$

(3) $$y=-(x^{\prime }-x_{0})\text{ sin }\theta^{\prime }+\;(y^{\prime }-y_{0})\;\cos\;\theta^{\prime }$$

Then, each receptive field was fitted with a family of spatial Gaussian and Gabor functions multiplied by a temporal Gaussian function:

**Table:** 

Function	Phase	Description	Equation
Gaussian Models	Monophasic	One spatial Gaussian with one temporal lobe	$RF=A_{s}*\exp\left(-\frac{x^{2}}{\sigma_{x}^{2}}-\frac{y^{2}}{\sigma_{y}^{2}}\right)*\exp\left(-\frac{{\left(t-\tau \right)}^{2}}{\sigma_{t}^{2}}\right)$
	Biphasic	Two spatial Gaussians each with one temporal lobe	$RF=A_{s1}*\exp\left(-\frac{x^{2}}{\sigma_{x1}^{2}}-\frac{y^{2}}{\sigma_{y1}^{2}}\right)*\exp\left(-\frac{{\left(t_{1}-\tau \right)}^{2}}{\sigma_{t1}^{2}}\right)+A_{s2}*\exp\left(-\frac{x^{2}}{\sigma_{x2}^{2}}-\frac{y^{2}}{\sigma_{y2}^{2}}\right)*\exp\left(-\frac{{\left(t_{2}-\tau \right)}^{2}}{\sigma_{t2}^{2}}\right)$
		One spatial Gaussian with two temporal lobes	$RF=A_{s}*\exp\left(-\frac{x^{2}}{\sigma_{x}^{2}}-\frac{y^{2}}{\sigma_{y}^{2}}\right)*\left(A_{t}*\exp\left(-\frac{{\left(t_{1}-\tau \right)}^{2}}{\sigma_{t1}^{2}}\right)-\exp\left(-\frac{{\left(t_{2}-\tau \right)}^{2}}{\sigma_{t2}^{2}}\right)\right)$
Gabor Models	Monophasic	One spatial Gabor with one temporal lobe	$RF=A_{s}*\exp\left(-\frac{x^{2}}{\sigma_{x}^{2}}-\frac{y^{2}}{\sigma_{y}^{2}}\right)*\;\cos\;\left(\frac{2\pi x}{\lambda }+\phi \right)*\exp\left(-\frac{{\left(t-\tau \right)}^{2}}{\sigma_{t}^{2}}\right)$
		One spatial Gabor with two cosine and one temporal lobe	$RF=A_{s}*\exp\left(-\frac{x^{2}}{\sigma_{x}^{2}}-\frac{y^{2}}{\sigma_{y}^{2}}\right)*\text{ cos }\left(\frac{2\pi x}{\lambda_{1}}+\phi_{1}\right)*\;\cos\;\left(\frac{2\pi y}{\lambda_{2}}+\phi_{2}\right)*\text{exp}\left(-\frac{{\left(t-\tau \right)}^{2}}{\sigma_{t}^{2}}\right)$
	Biphasic	One spatial Gabor with two temporal lobes	$RF=A_{s}*\exp\left(-\frac{x^{2}}{\sigma_{x}^{2}}-\frac{y^{2}}{\sigma_{y}^{2}}\right)*\;\cos\;\left(\frac{2\pi x}{\lambda }+\phi \right)*\left(A_{t}*\exp\left(-\frac{{\left(t_{1}-\tau \right)}^{2}}{\sigma_{t1}^{2}}\right)-\exp\left(-\frac{{\left(t_{2}-\tau \right)}^{2}}{\sigma_{t2}^{2}}\right)\right)$
		One spatial Gabor and one spatial Gaussian each with a temporal lobe	$RF=A_{s}*exp\left(-\frac{x^{2}}{\sigma_{x1}^{2}}-\frac{y^{2}}{\sigma_{y1}^{2}}\right)*\text{ cos }\left(\frac{2\pi x}{\lambda }+\phi \right)*\exp\left(-\frac{{\left(t_{1}-\tau \right)}^{2}}{\sigma_{t1}^{2}}\right)+exp\left(-\frac{x^{2}}{\sigma_{x2}^{2}}-\frac{y^{2}}{\sigma_{y2}^{2}}\right)*\exp\left(-\frac{{\left(t_{2}-\tau \right)}^{2}}{\sigma_{t2}^{2}}\right)$

### Temporal analysis

To interpret the neural response to the Gaussian full field flash stimulus, a linear non-linear cascade was used which modeled the cellular response by first applying linear filters to the stimulus and then passing the result through a static non-linearity. Specifically, the model is given by:

(4) $$r_{est}\left(t\right)=G\left(f_{1}\;*\;s,f_{2}\;*\;s,\cdots \right)$$

where **f**_*i*_ is a set of linear filters, **s** is the stimulus and *G* is the static non-linearity.

The linear filter set was obtained by analyzing the statistics of the ensemble of the stimulus before the spike. Specifically, we used STA and spike-triggered covariance (STC) analysis by first calculating the STC matrix:

(5) $$STC=\frac{1}{N_{spikes}-1}\sum_{n=1}^{N_{spikes}}\left[s\left(t_{n}\right)-STA\right]{\left[s\left(t_{n}\right)-STA\right]}^{T}\;$$

where:

(6) $$STA=\frac{1}{N_{spikes}}\sum_{n=1}^{N_{spikes}}s\left(t_{n}\right)$$

*N*_*spikes*_ denotes the number of spikes and ***s***(t_n_) is the stimulus presented over some fixed time interval preceding the *n*-th spike. Then, the eigenvectors of the STC matrix were calculated and together with the STA were used as linear filters. Generally, only the STA and/or the eigenvectors with the highest or lowest corresponding eigenvalues associated with excitatory and inhibitory dimensions were found to be part of a significant linear non-linear model. To determine whether a filter was a part of a significant model or not, the model performance was compared to the chance level. To obtain the chance level, first a set of linear non-linear models based on the intermediate eigenvectors (eigenvectors 5–10), as associated with insignificant models, was generated. Then the correlation coefficients were calculated between these models and the true neuronal firing patterns. Last the critical threshold was set as the mean plus five standard deviations of the resulting correlation coefficients of the population.

## Results

### Spatiotemporal receptive fields

**Figure 2:**
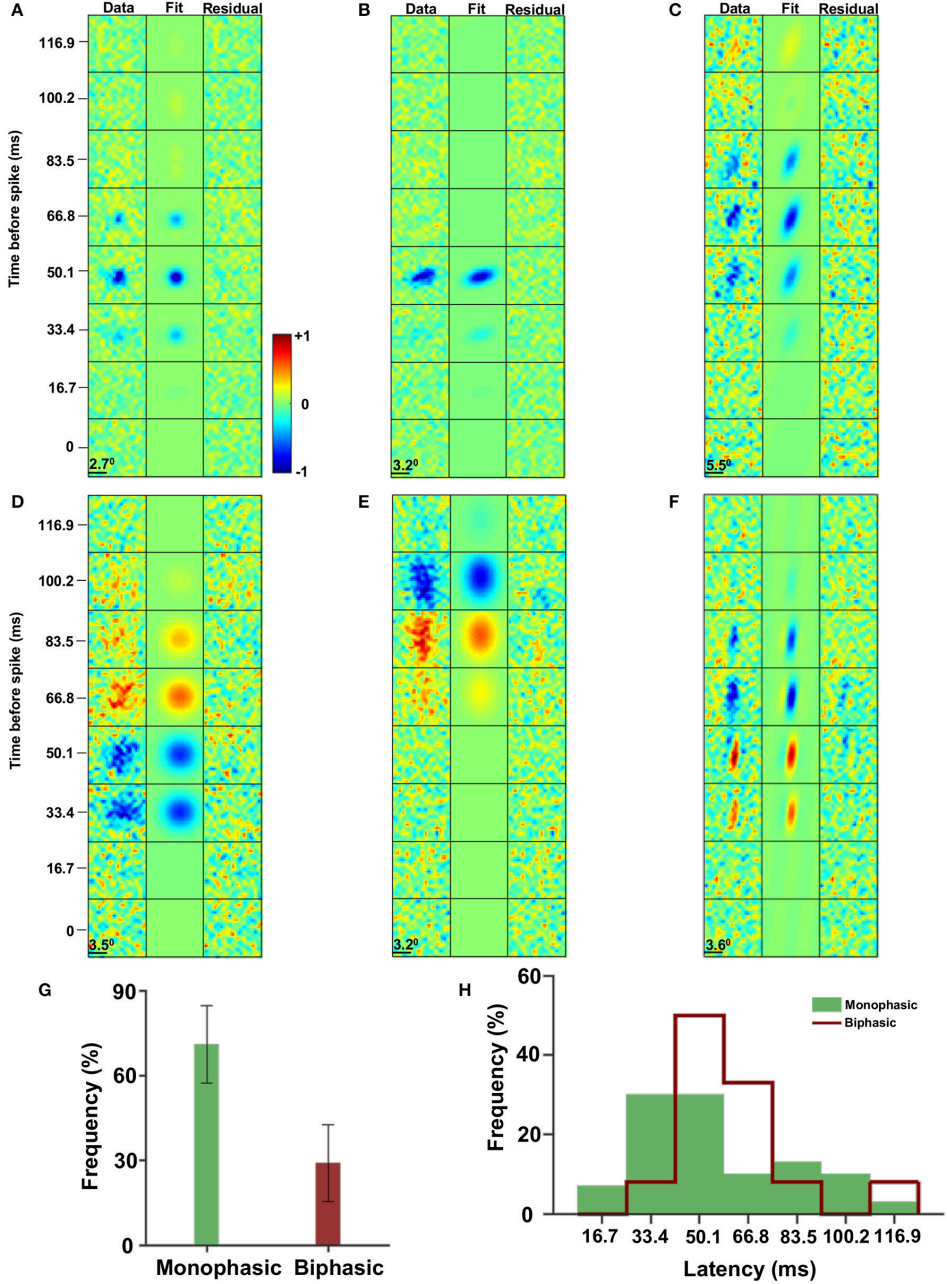
Spatiotemporal receptive fields. **(A–F)** The spatiotemporal receptive fields are characterized by temporal monophasic **(A–C)** and biphasic **(D–F)** leading to low pass filter **(A–C)** and band pass filter **(D–F)** groups. In addition the eccentricity of the receptive field spatial profile has a broad distribution from nearly circular **(A,D)** to highly elliptic **(C,F)**. **(G)** The distribution of monophasic (~2/3) and biphasic (~1/3) receptive fields (with a 95% confidence interval). **(H)** The distribution of the STA latency response of monophasic (green; 54.5 ± 26 ms, mean ± STD) and biphasic (red line; 60 ± 21 ms, mean ± STD) receptive fields (*p* = 0.73, permutation test).

To reveal the space-time structure of the receptive fields in the archerfish optic tectum, neurons localized in the superficial layers of the optic tectum were recorded using a single extracellular electrode. Each cell was stimulated by a random checkerboard stimulus (see Material and Methods) and the spatiotemporal spike-triggered average (STA) was calculated, where the maximum absolute value over space and time was normalized to one (Figures 2A–F). In some cases the STA did not reveal an above-noise level structure (21 out of 63) resulting in a dataset of *n* = 42 cells.

The majority of visual receptive fields of the cells in the optic tectum are characterized by two general features. The first is the eccentricity of the spatial profile of the receptive field. Specifically, the receptive field shapes ranged from almost circular (Figures 2A,D) to slightly elliptical (Figures 2B,E) to highly elliptical (Figures 2C,F).

The second feature is related to the differences in the temporal profile of the receptive fields, which fell into two broad cell groups. One group could be characterized by its monophasic profile with an OFF time lobe alone (Figures 2A–C). The other group was termed an ON/OFF group since its receptive fields had a biphasic response profile in time (specifically 70% ON/OFF and 30% OFF/ON). That is, the receptive fields had both positive (i.e., a high light level before the spike) and negative (i.e., a low light level before the spike) STA values (Figures 2D–F). The monophasic group constituted the majority of the population (68%, Figure 2G). In addition, the monophasic group had a slightly shorter response latency (54.5 ± 26 ms, mean ± STD) compared to the biphasic group (60 ± 21 ms, mean ± STD; Figure 2H; *p* = 0.73, permutation test). Defining the size as *S*_*RF*_ = σ_*x*_ · σ_*y*_, both groups had similar sizes (ON/OFF; mean_ON/OFF_ = 5.7 degrees, STD_ON/OFF_ = 2.3 degrees, OFF: mean_OFF_ = 5.6 degrees, STD_OFF_ = 2.8 degrees).

To analyze the spatiotemporal structure of the receptive fields and the eccentricity in particular, the data were fitted with three different spatial Gaussian and four different spatial Gabor functions multiplied by temporal Gaussian functions, and the parameters of the best fit were used (see Material and Methods). The results showed that generally the Gaussian models outperformed the Gabor based models (37 out of 42 cells).

**Figure 3:**
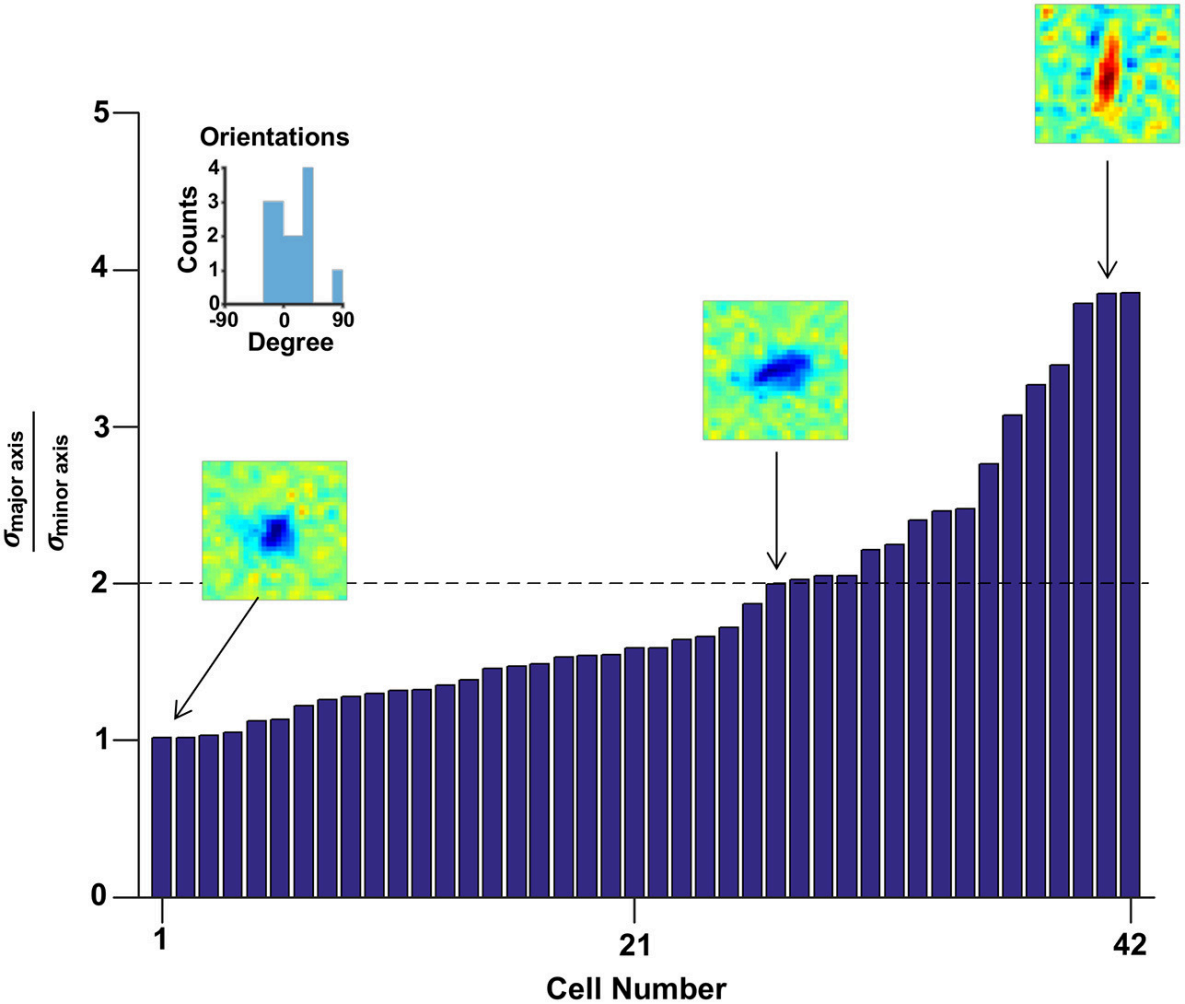
The distribution of eccentricities. The shape of the receptive field varied from round to elongate as shown in the ratio of the major to the minor intrinsic axes of the receptive fields. The orientations of the receptive fields which met the criterion of $\frac{\sigma_{major\;axis}}{\sigma_{minor\;axis}}\geq 2$ (*n* = 15) are presented in the inset frame.

Then the best fitted model was used to quantify the eccentricity distribution by calculating the ratio of the major axis to the minor axis of the receptive fields (Figure 3). The ratios varied from roughly circular receptive fields with $\frac{\sigma_{major\;axis}}{\sigma_{minor\;axis}}≅1$ to elongated ellipses up to $\frac{\sigma_{major\;axis}}{\sigma_{minor\;axis}}=3.8$. At a dividing line of 2 to distinguish between low and high eccentricities, 64% had low eccentricities and 36% had high eccentricities. Furthermore, the orientation of the elongated receptive fields $\left(\frac{\sigma_{major\;axis}}{\sigma_{minor\;axis}}\geq 2,\;n=15\;cells\right)$ were spread around 0 degrees (i. e., a vertical ellipse, inset of Figure 3).

### Recovering the temporal linear filters underlying cellular function

To better understand the information processing by archerfish optic tectum cells, the possibility that multiple filters contribute to cellular activity was explored. Random checkerboard stimulus has the disadvantage of large number stimulus dimensions, which makes it difficult to estimate additional linear filters beyond the STA. To overcome this difficulty, a full field flash with Gaussian statistics was used since it is a function of time alone and hence is amenable to further analysis.

**Figure 4:**
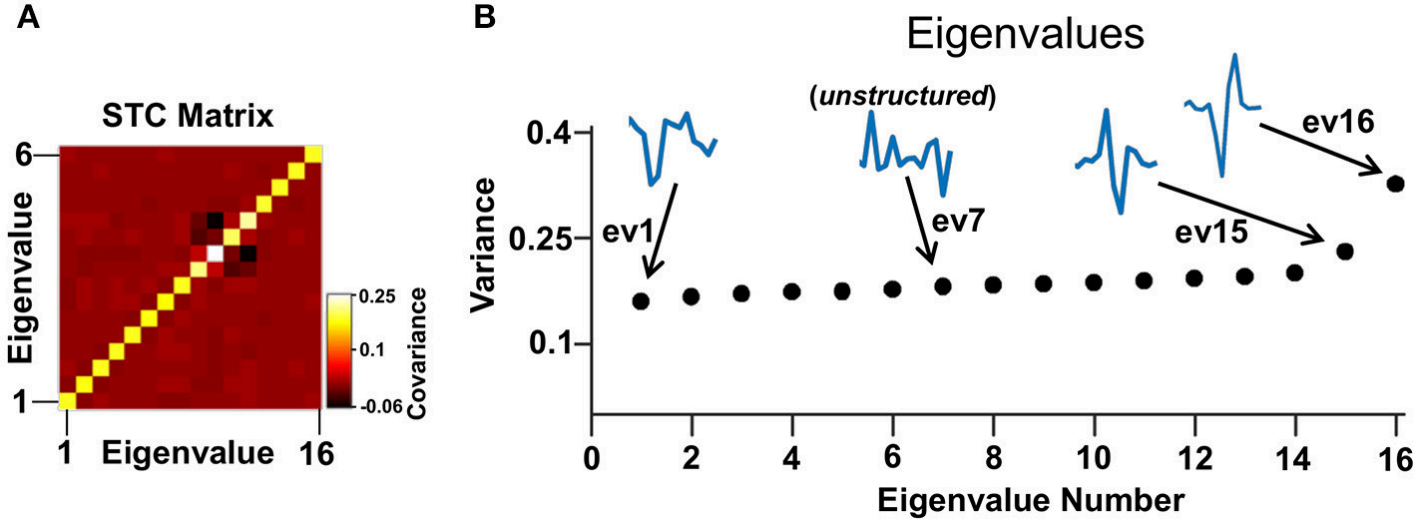
STC analysis. **(A)** An example of a STC matrix of a representative cell. **(B)** The eigenvalues of the STC matrix in **(A)**. In this example there are two excitatory and one inhibitory eigenvalues. Their associated eigenvectors have a defined shape in contrast to a noise level eigenvector that has an arbitrary shape (e.g., eigenvector 7).

It was assumed that each cell could be described as a linear non-linear Poisson model where a set of linear filters are applied to the stimulus and can be combined to obtain the neuronal rate by an instantaneous non-linearity (Hunter and Korenberg, 1986; Meister and Berry, 1999; Chichilnisky, 2001; Keat et al., 2001; Paninski, 2003; Rust et al., 2004, 2005; Carandini et al., 2005; Rust and Movshon, 2005; Schwartz et al., 2006; Beaudoin et al., 2007; Horwitz et al., 2007; Pillow et al., 2008; Solomon et al., 2010; Ostojic and Brunel, 2011; Estebanez et al., 2012; Samengo and Gollisch, 2013; Vasserman et al., 2013; Tkačik et al., 2014; Sandler and Marmarelis, 2015). Then, a Poisson generator converted the firing rate signal into spikes. Based on the length of the STA the filters were set to operate within 250 ms before the spike. The ensemble of stimulus intervals preceding each spike defined the spike-triggered stimulus ensemble. Then, using spike-triggered covariance (STC) analysis the set of linear filters that described the cellular function was recovered (Schwartz et al., 2006). The first filter was defined as the STA; i.e., the mean of the spike-triggered stimulus ensemble. Additional filters were obtained by finding the directions in the stimulus space where the variance was greater than expected by chance. This was done by first calculating the covariance matrix of the STC ensemble (Figure 4A) and then using principal component analysis to obtain the directions in space associated with an increase (e.g., eigenvalues 15 and 16, Figure 4B) or a decrease (e.g., eigenvalue 1, Figure 4B) in variance as compared to the one expected by chance (Rust et al., 2004, 2005; Carandini et al., 2005; Schwartz et al., 2006; Horwitz et al., 2007; Solomon et al., 2010; Estebanez et al., 2012; Samengo and Gollisch, 2013; Sandler and Marmarelis, 2015). Using the terminology introduced in Rust et al. (2005), excitatory and inhibitory filters were defined based on whether their associated eigenvalue was larger or smaller than the variance of the raw stimulus (e.g., Figure 4B, eigenvalues 1, 15, and 16). The excitatory or inhibitory filters had a structured shape (e.g., Figure 4B, eigenvectors 1, 15, and 16) whereas the remaining filters had a random shape fluctuating around zero (e.g., Figure 4B, eigenvector 7).

### Optic tectum cells are characterized by several linear filters

The STA and each of the excitatory and inhibitory filters were used separately to fit a one-dimensional linear non-linear model to the neural response. The rationale for fitting sequentially single filter models to the data was to find which stimulus dimensions were linked to the cellular response function. Subsequently, these filters were used to fit a full (possible multidimensional) model to the neuronal response.

**Figure 5:**
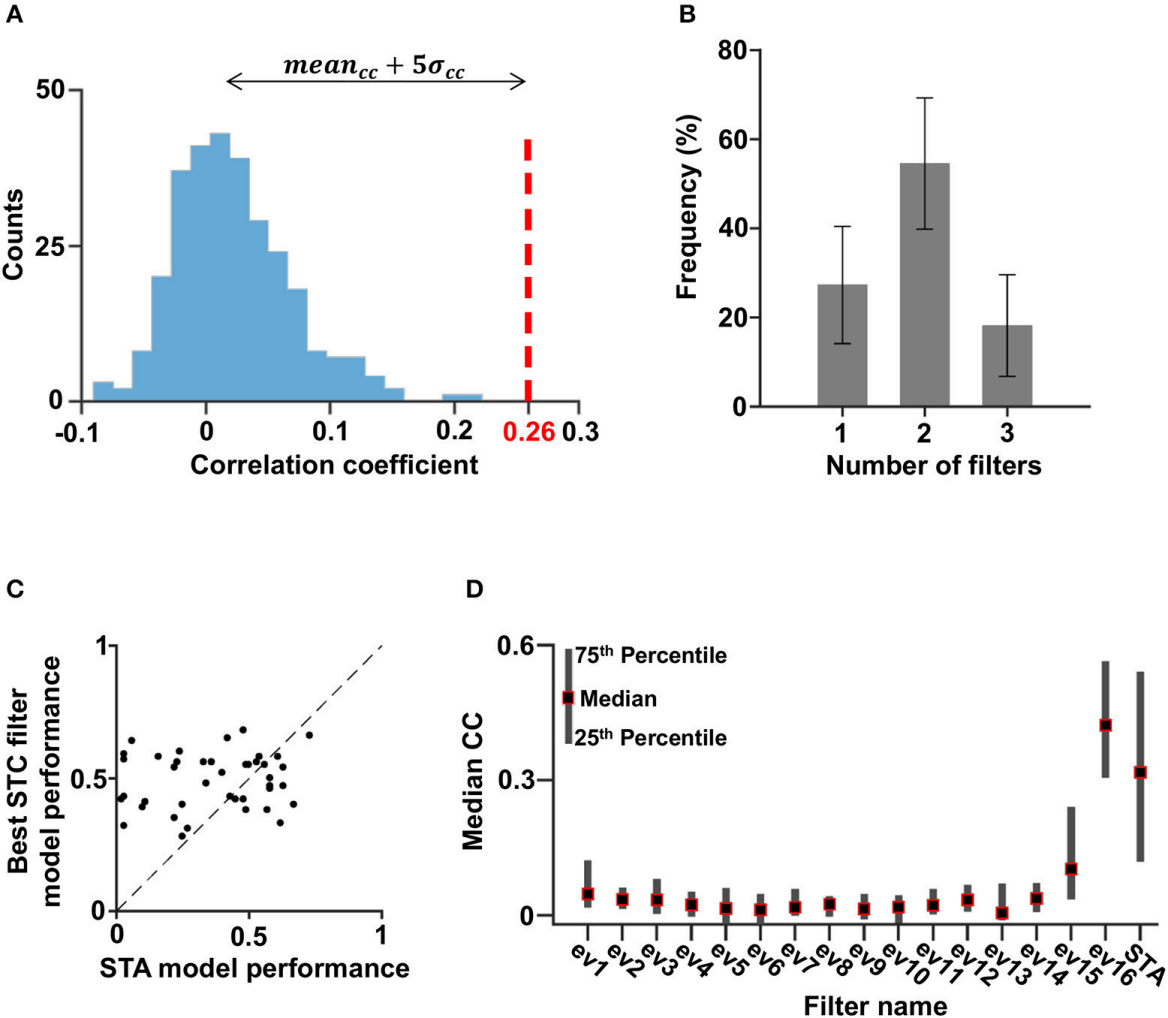
Filters of significant models. **(A)** The distribution of the correlation coefficients obtained with eigenvectors 5–10 of all cells. The value of the mean plus five times the standard deviation was set as the cutoff such that models associated with correlation coefficients above this number were considered significant (red striped line). **(B)** The distribution of the number of filters per cell (with a 95% confidence interval). Most cells had two filters. **(C)** The correlation coefficient associated with the STA filter vs. the best correlation coefficient associated with one of the STC filters. For 36% of the cells, the highest correlation coefficient was obtained by using the STA filter, and for 64% of the cells, the highest correlation coefficient was obtained by using an excitatory filter. Thus, in most cases the contribution of the STC filter was more significant; hence, the STC analysis made a good contribution to this kind of model. **(D)** The one-dimensional linear non-linear model correlation coefficient median of all cells.

To determine the relevant dimensions for each cell, the success of a single filter model was quantified by calculating the Pearson correlation coefficient (Carandini et al., 2005; Chen et al., 2007; Ostojic and Brunel, 2011) between the model prediction and the neuronal firing rate. A filter was said to be linked to the cell functional properties if the correlation coefficient was significantly above noise level. In our case this threshold criterion yielded 0.26 (Figure 5A, see Material and Methods). This definition of threshold criterion was compared with the significance test presented in Rust et al. (2005) and Schwartz et al. (2006) by comparing the significant models determined by each method, and was found to be more strict. The cells could be described by one to three filters, with the majority characterized by two filters (Figure 5B). It should be noted that models based on a single filter obtained from the STC analysis generally achieved a higher predictive power than models based on the STA (Figure 5C).

Some cells may have had additional filters that could not pass the criterion due to lack of sufficient data. However, the stimulus dimensions associated with such filters; i.e., filters based on eigenvectors 5–10, only made a marginal contribution to cellular activity as revealed by the low correlation with the neuronal response of such models (Figure 5D).

### The structure of multidimensional non-linearities are characterized by several different fundamental shapes

After the set of linear filters that characterized each cell were determined, the multidimensional non-linearities that linked the filter outputs and the predicted firing rate were calculated. This was done by matching the firing rates and the filter outputs for a training dataset i.e., the repeated stimulus intervals. This procedure was feasible for cells with up to three filters. Overall the performance of the multi filter model was significantly better than the STA filter model (paired *t*-test *t*_30_ = 2.54, *p* < 0.05, $x_{multi}\;=\;0.52,\;x_{STA}\;=\;0.46$).

**Figure 6:**
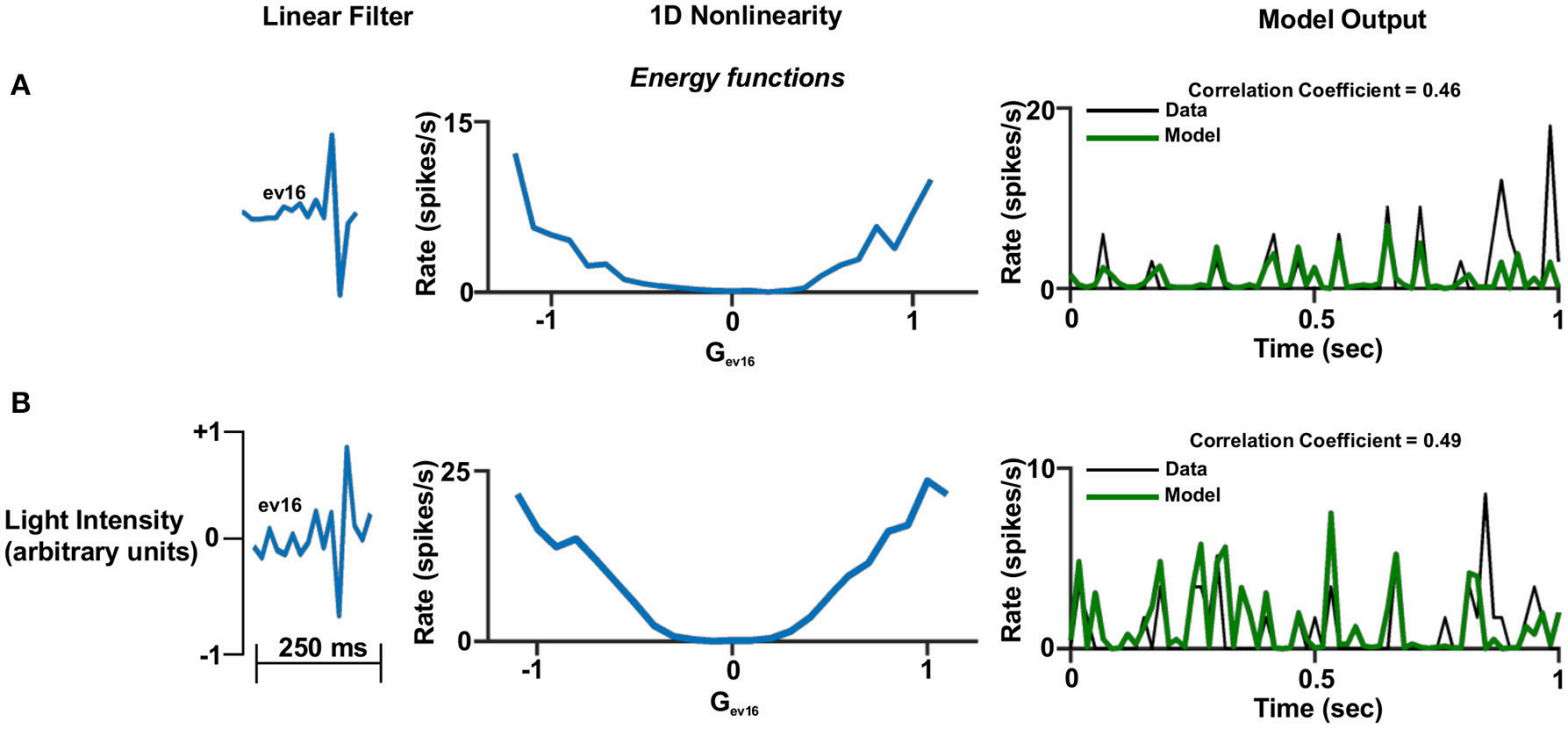
One dimensional linear non-linear model. **(A,B)**. One dimensional linear non-linear models of two different cells. Left panel: The linear filter. Middle panel: The non-linear function where the generator units are normalized. A constant was added to the function in order for its minimum to be set to zero. This is legitimate since it does not affect the correlation coefficient. Right panel: One second of the real firing rate vs. the model's firing rate prediction. The model's firing rate was multiplied by a constant in order for its amplitude to match the true firing rate. This is legitimate since it does not affect the correlation coefficient.

**Figure 7:**
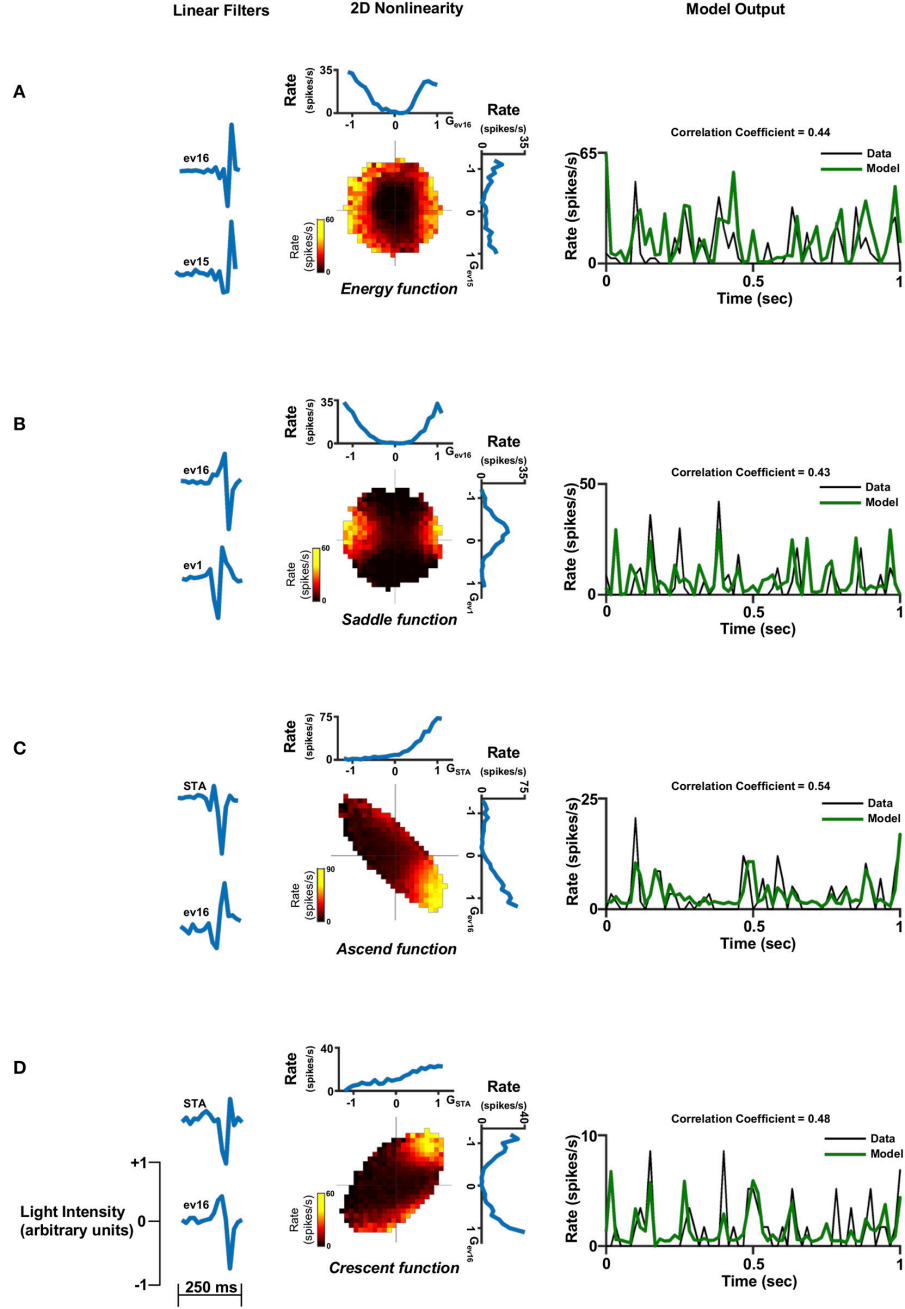
Two dimensional linear non-linear model. Two dimensional linear non-linear models of four different cells representing the four non-linear function shapes: **(A)** Energy function. **(B)** Saddle function. **(C)** Ascend function and **(D)** Crescent function. Left panel: The two linear filters. Middle panel: The non-linear function where the generator units are normalized. A constant was added to the function in order for its minimum to be set to zero. This is legitimate since it does not affect the correlation coefficient. Right panel: One second of the real firing rate vs. the model's firing rate prediction. The model's firing rate was multiplied by a constant in order for its amplitude to match the true firing rate. This is legitimate since it does not affect the correlation coefficient.

**Figure 8:**
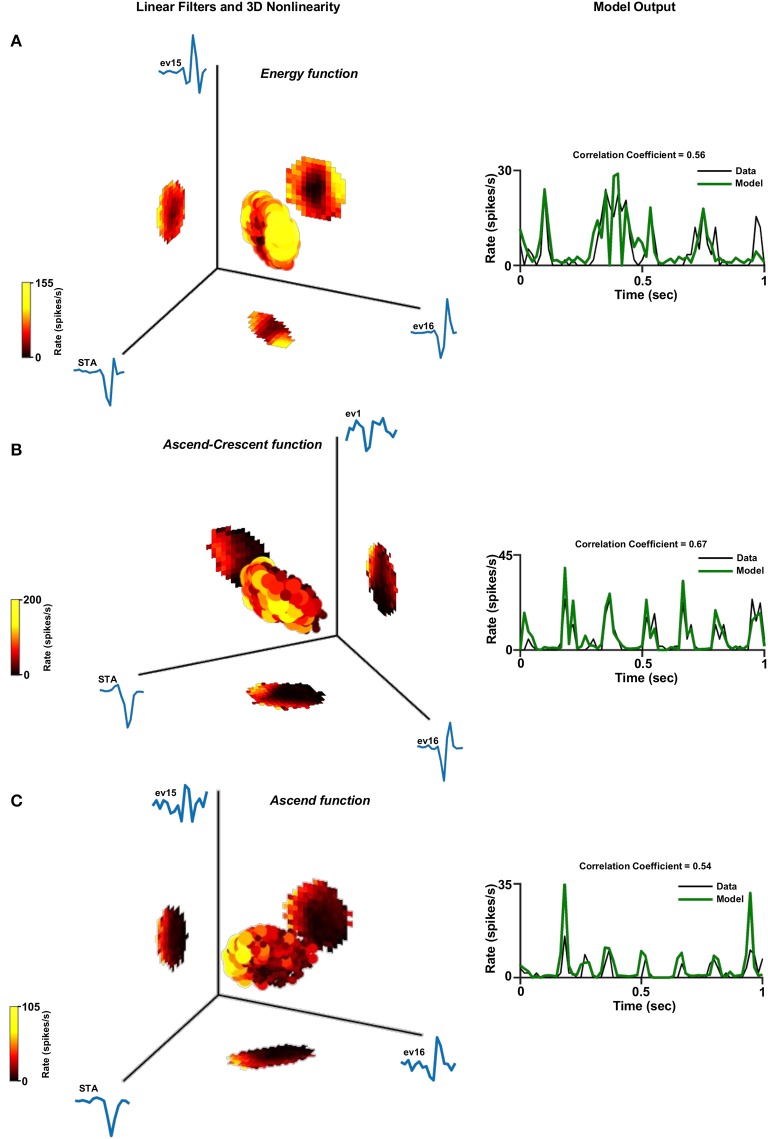
Three dimensional linear non-linear model. Three dimensional linear non-linear models of three different cells: **(A)** Energy function. **(B)** Ascend-Crescent function and **(C)** Ascend function. Left panel: The non-linear function where the generator units are normalized. A constant was added to the function in order for its minimum to be set to zero. This is legitimate since it does not affect the correlation coefficient. Right panel: One second of the real firing rate vs. the model's firing rate prediction. The model's firing rate was multiplied by a constant in order for its amplitude to match the true firing rate. This is legitimate since it does not affect the correlation coefficient.

Figures 6–**8** show examples of the non-linearities for cells that could be characterized by one, two or three stimulus dimensions. The presentations of the structure of the multidimensional non-linearities used heat maps. In addition, the one/two dimensional projections of each filter that contributed to a multidimensional non-linearity are presented.

In general, the one-dimensional non-linearity (or the one-dimensional projection) had one of three basic shapes: (1) An energy model shape; i.e., firing rate functions that increased monotonically with the magnitude of the generator (Figures 6A,B, 7A,B,D, 8A,B), (2) A hill shape; i.e., firing rate functions that decreased monotonically with the magnitude of their generator (Figure 7B), (3) A half-wave rectified shape (Figures 7C,D, 8B,C). These functional types are traditionally assigned to the temporal domain of simple and complex cells in the primary visual cortex (V1).

The non-linear projections obtained from the excitatory filters were dominated by the energy function shape (56% of the filter population, *n* = 44) and the minority had a half-wave rectified (40% of the filter population, *n* = 31) shape. This contrasted with the inhibitory filters which mostly had a hill shape (66% of the filter population, *n*_Hill_ = 2) and fewer had a half-wave rectified shape (34% of the filter population, *n*_HWR_ = 1).

Examination of the population of cells characterized by two filters, which was the majority of the cell population (Figure 5B), revealed that the two-dimensional non-linear function had one out of four different shapes: The energy function where its one dimensional projections both had the energy shape (Figure 7A); saddle function where its one dimensional projections had the energy and hill shapes (Figure 7B); ascend function where its one dimensional projections had both the half-wave rectified shape (Figure 7C); or Crescent function where its one dimensional projections had the energy and half-wave rectified shapes (Figure 7D). Similar structures can be seen in the few cells that were characterized by three filters (Figure 8).

## Discussion

The purpose of this study was to explore the spatiotemporal structure of the receptive field of cells in the archerfish optic tectum. The spatiotemporal linear filter was measured by calculating the STA in response to random checkerboards. Receptive field properties such as size, eccentricity, temporal phase, and response latency were defined. The results showed that the receptive field's spatiotemporal structure mostly fit a two-dimensional spatial Gaussian multiplied by a temporal monophasic or biphasic part. The eccentricities of the spatial Gaussians varied from 1 to 3.8, thus ranging from a circular shape to an ellipsoid shape respectively.

**Figure 9:**
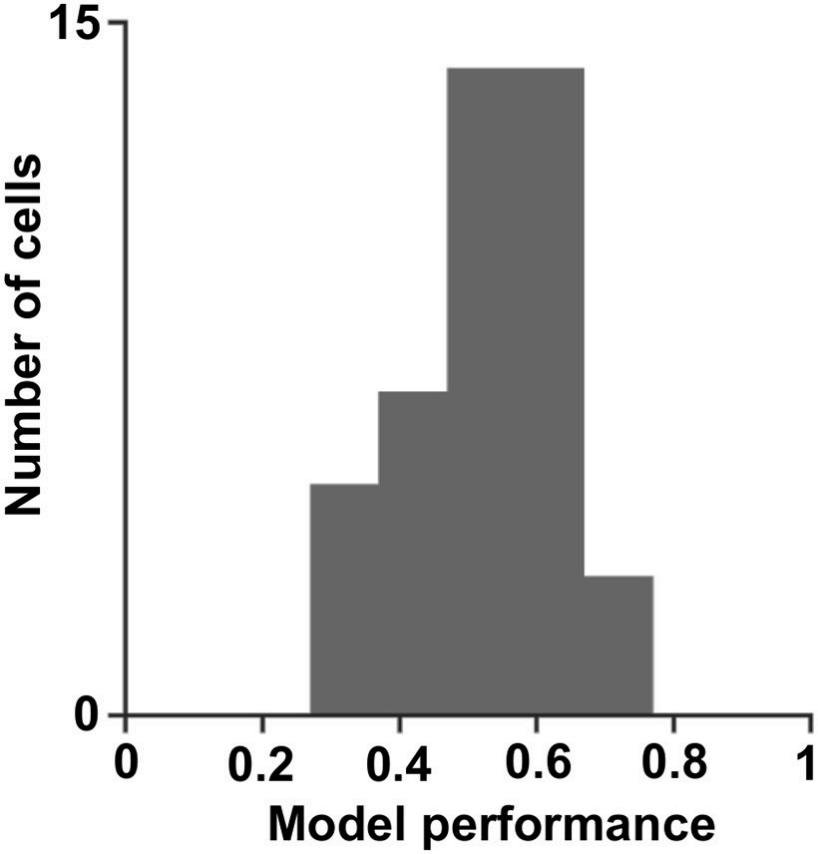
Model performance. A histogram of the correlation coefficients obtained by fitting the linear non-linear model to the cell's true response (0.5 **±** 0.1; mean **±** STD).

The temporal neural activity was characterized by fitting linear non-linear models to the data to predict the cell's response to arbitrary stimuli. We used the differences in the structure of the model, either in the filters shape or the structure of the non-linear response to reveal the coding scheme of each neuron. The results showed that cells could be described by one to three dimensional models, and their overall performance mean was 0.5 ± 0.1 (mean ± STD, significantly more than chance, *p* = 0, permutation test; Figure 9). All cells had excitatory filters and only a few had inhibitory filters. The one-dimensional non-linear function had one of three fundamental shapes, of which the energy function shape was the most prevalent (56%). The other two shapes were the half-wave rectified and hill functions (40 and 4% of the cells, respectively).

Theoretical frameworks have demonstrated that the environment's statistics influences the visual receptive fields and attempted to predict the receptive fields of terrestrial mammals (Field, 1987; Olshausen and Field, 1996; van Hateren and van der Schaaf, 1998). Since the archerfish's visual systems needs to process on a daily basis two environments that differ in their statistical properties (Balboa and Grzywacz, 2003), one may naively assume that the archerfish might have more than one population of receptive fields, each devoted to different visual environment. However, our results do not indicate the existence of two unique populations of receptive fields. The receptive field's eccentricity lie on a continuum (Figure 3) and we do not observe significantly more than one function that fits the spatial component of the receptive fields.

There are several possible explanations that can lead to this result. First, the differences between terrestrial and underwater statistics are too small. Thus, the pressure to evolve two distinct receptive field populations is weak. Second, theoretical framework assumptions are incomplete, that is, the assumptions underlie the theory should be modified. In fact, there is indication that the assumptions are incomplete since even when considering results from mammalian early visual system, such discrepancy between receptive fields obtained experimentally and theoretically was presented. Specifically, the spatial profile of receptive fields recorded from macaque and cat primary visual cortex differ from the predicted theoretical receptive field structures (Ringach, 2002).

The use of both checkerboard and Gaussian white noise stimuli was necessary in studying the cellular functional properties in this study. The main reason is due to the fact that the checkerboard stimulus has too many dimensions that make the STC analysis infeasible since it is difficult to estimate additional linear filters beyond the STA. Thus, we adopted the approach of measuring the spatial profile of the cell using checkerboard and temporal profile using full field Gaussian white noise. Alternative approaches to address this issue are to reduce the number of checkers using one or two optimal time frames (Chen et al., 2007). Another approach to overcome this problem is using binary white noise stripes instead of checkers (Rust et al., 2005). While these methods are useful in reducing dimensionality, they lack the ability to extract a full spatiotemporal description of the cells. Since our goal here was to study the spatiotemporal cellular properties, the checkerboard stimulus was kept and an additional temporal stimulus was interleaved with it.

### Comparison with other descriptions of receptive field properties in the fish tectum

Since the optic tectum is the main visual processing unit in fish (Northmore, 2011), many studies have been devoted to it. However, most research on the optic tectum's visual cells has focused on their receptive field classification in terms of direction selectivity. Studies on the archerfish (Ben-Tov et al., 2013), goldfish (Maximov et al., 2005), and zebrafish (Niell and Smith, 2005; Johnston and Lagnado, 2012) have shown that the visual receptive fields can be classified into three categories: orientation-tuned cells, direction-tuned cells, and direction-agnostic cells. Other research was devoted to investigate the functionality of different layers of the optic tectum (Del Bene et al., 2010). However, no full investigations of the spatiotemporal structure using white noise analysis have been conducted (Ramdya et al., 2006). The current study used white noise analysis and found that the optic tectum cells' receptive fields are characterized by temporal phase, eccentricity, delay time and shape. These findings highlight the importance of combining STC analysis with the STA filter since the non-linearity can be used to classify the cells into simple and complex cells as is traditionally done for the primary visual cortex cells of mammals. Moreover, using moving bar stimulus, most cells were agnostic in direction. For simple cells, 44% were direction selective and 56% direction agnostic (*n* = 9) and for complex cells, 30% were direction selective and 70% direction agnostic (*n* = 17). These insights thus broaden our knowledge of the optic tectum's functionality in fish.

### Comparison to non-mammalian vertebrates' optic tectum spatial receptive field structures

In many non-mammalian vertebrates, the superficial layers of the optic tectum topographically receive direct input from the retina and respond in particular to visual stimuli (Novales Flamarique and Wachowiak, 2015). It is the main visual processing unit and in some vertebrates including fish it is one of the largest and most significant components of the brain. The optic tectum is involved in the deployment of spatial attention and the orientation of movements. The spatial structure of the optic tectum's receptive fields of non-mammalian vertebrates such as amphibians (Gaillard and Galand, 1977), birds (Verhaal and Luksch, 2013), and reptiles (Stein and Gaither, 1983) have a Gaussian-like shape varying from circles to elongated ellipses. This is in partial agreement with the findings here since some of the archerfish optic tectum cells had a Gabor-like receptive field.

### Comparison to terrestrial mammals' spatial receptive field structures

The straightforward approach consists of comparing the archerfish optic tectum visual region to the superior colliculus of terrestrial mammals since they are homologs. Studies in the rat (Humphrey, 1968), monkey (Churan et al., 2012), and mouse (Wang et al., 2010; Feinberg and Meister, 2015) have shown that the shapes of the receptive field in the superior colliculus of mammals are round, which may serve mainly for target position representation. Since these types of receptive fields are only part of what was found in the archerfish optic tectum, the comparison of non-mammalian optic tecta to the mammalian superior colliculus is incomplete.

An alternative approach would involve comparing the two main visual processing units of the species. The main visual processing unit of terrestrial mammals is the primary visual cortex (V1) where a broad, thorough investigation of the cellular receptive fields structures has been conducted on a variety of terrestrial mammals such as the monkey (Ringach, 2002), cat (Hubel and Wiesel, 1962; Jones and Palmer, 1987a,b), rat (Girman et al., 1999), mouse (Niell and Stryker, 2008), and ferret (Usrey et al., 2003). All these species share the same receptive field features which extend from symmetrical circles to elongated ellipses with excitatory and inhibitory sub-regions. The latter are commonly represented by a Gabor function, possibly to serve as an edge detector. This idea complements the findings here since any Gabor function is simply a combination of Gaussians.

Support for similar functionality of the two brain regions comes from Zhaoping's comparison of the optic tecta of non-mammalian vertebrates to the visual cortex of mammals, which suggested that both create a saliency map from visual input (Zhaoping, 2016). Therefore it was hypothesized that the saliency map migrated over the course of evolution from the superficial layers of the optic tectum to the primary visual cortex.

### Simple and complex cells in non-mammalian vertebrates and terrestrial mammals

One longstanding tradition is to divide the primary visual cortex of terrestrial mammals into simple and complex cells. These have been reported to exist in many mammals such as the monkey (Hubel and Wiesel, 1968; Ringach, 2002; Chen et al., 2007), cat (Hubel and Wiesel, 1962; Jones and Palmer, 1987a,b; Touryan et al., 2002; Felsen et al., 2005), rat (Estebanez et al., 2012), ferret (Usrey et al., 2003), and tree shrew (Van Hooser et al., 2013).

The distinction between simple and complex cells is largely based on the non-linear function that links the linear filter outputs to the firing rate. The non-linearity of simple cells has a half-wave rectified shape whereas the non-linearity of complex cells has an energy function shape. This served as the criterion in this study as well. Even though simple and complex cells have been thoroughly investigated and characterized in terrestrial mammals, this study is the first to have observed them in other vertebrates.

Specifically, the results showed that the three, non-linear energy, half-wave rectified and hill function types found in terrestrial mammals (Rust et al., 2004, 2005) were present in the archerfish as well. Hence, the current results are very similar to the findings obtained from area 17 of the cat, where white noise analysis of complex cells produced only two excitatory filters for most cells and no suppressive filters (Touryan et al., 2002). The results are also akin to the findings obtained from the primary visual cortex of monkeys with the minor caveat that in the latter more linear filters were obtained per cell (Rust et al., 2005). This could have been due to sampling issues since the number of spikes has a considerable influence on the number of filters revealed by STC analysis (Schwartz et al., 2006).

All the above suggests that even though the optic tectum is homologous to the superior colliculus of mammals, it might be functionally analogous to both the superior colliculus and the primary visual cortex. In other words, since the optic tectum of non-mammalian vertebrates is the main visual center and one of the largest components of its brain, it implements a wide variety of functions that exist in terrestrial mammals' basic visual brain regions.

### Simple and complex cells in different sensory modalities

The traditional division into simple and complex cells in the primary visual cortex has been extended to other sensory modalities. For example, somatosensory simple and complex cells have been recorded from the barrel cortex when responding to spatiotemporal patterns of stimuli applied to rat whiskers (Estebanez et al., 2012). In the primary auditory cortex of Rhesus monkeys, two types of cells with characteristics similar to visual simple and complex cells were found (Tian et al., 2013). The auditory simple cells had segregated sub-regions in frequency space where an “On” sub-region corresponded to a high firing rate when switching on a tone in a specific frequency sub-region and low firing rate when switching off a tone in the same frequency sub-region. “Off” sub-regions acted the opposite way from “On” sub-regions and were adjacent in frequency space to the “On” sub-regions. Complex auditory cells had “On” and “Off” regions that overlapped. The existence of similar types of receptive fields in the different sensory regions of the cortex and their existence in aquatic and terrestrial vertebrates may thus hint at the existence of a universal canonical processing algorithm for sensory information.

## Author contributions

AR: designed study, performed experiments, analyzed data, and wrote the manuscript; MB-T: designed study, performed experiments, analyzed data, and revised the manuscript; RS: designed study, analyzed data, and wrote the manuscript.

### Conflict of interest statement

The authors declare that the research was conducted in the absence of any commercial or financial relationships that could be construed as a potential conflict of interest.
